# Fast Growth of Highly Ordered TiO_2_ Nanotube Arrays on Si Substrate under High-Field Anodization

**DOI:** 10.1007/s40820-016-0114-4

**Published:** 2016-11-09

**Authors:** Jingnan Song, Maojun Zheng, Bin Zhang, Qiang Li, Faze Wang, Liguo Ma, Yanbo Li, Changqing Zhu, Li Ma, Wenzhong Shen

**Affiliations:** 1 Key Laboratory of Artificial Structure and Quantum Control, Ministry of Education, Department of Physics and Astronomy, Shanghai Jiao Tong University, Shanghai, 200240 People’s Republic of China; 2 Collaborative Innovation Center of Advanced Microstructures, Nanjing, 210093 People’s Republic of China; 3 Institute of Fundamental and Frontier Sciences, University of Electronic Science and Technology of China, Chengdu, 610054 People’s Republic of China; 4 School of Chemistry and Chemical Technology, Shanghai Jiao Tong University, Shanghai, 200240 People’s Republic of China

**Keywords:** TiO_2_ nanotube arrays, Si substrate, Anodization, High field, Controllable preparation

## Abstract

**Abstract:**

Highly ordered TiO_2_ nanotube arrays (NTAs) on Si substrate possess broad applications due to its high surface-to-volume ratio and novel functionalities, however, there are still some challenges on facile synthesis. Here, we report a simple and cost-effective high-field (90–180 V) anodization method to grow highly ordered TiO_2_ NTAs on Si substrate, and investigate the effect of anodization time, voltage, and fluoride content on the formation of TiO_2_ NTAs. The current density–time curves, recorded during anodization processes, can be used to determine the optimum anodization time. It is found that the growth rate of TiO_2_ NTAs is improved significantly under high field, which is nearly 8 times faster than that under low fields (40–60 V). The length and growth rate of the nanotubes are further increased with the increase of fluoride content in the electrolyte.

**Graphical Abstract:**

Highly ordered TiO_2_ nanotube arrays (NTAs) on Si substrate have been fabricated by high-field anodization method. A high voltage (90–180 V) leads to a high growth rate of TiO_2_ NTAs (35–47 nm s^−1^), which is nearly 8 times faster than the growth rate under low fields (40–60 V). Furthermore, the current density–time curves recorded during the anodization provide a facial method to determine the optimal anodization parameters, leading to an easy obtaining of the desired nanotubes.
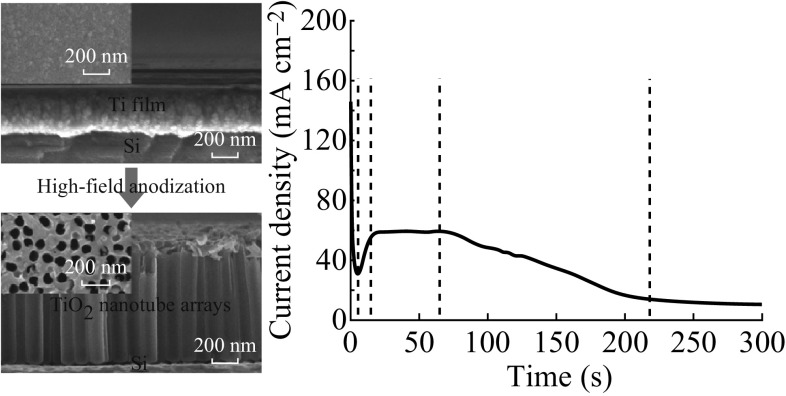

## Introduction

Since the first report on the growth of highly ordered TiO_2_ nanotube arrays (NTAs) by a simple electrochemical anodization method in 1999 [1], TiO_2_ NTAs prepared by electrochemical anodization of titanium foil and alloys have attracted much attention in various applications, such as corrosion protection [2, 3], sensors [4–6], dye-sensitized solar cells [7], photocatalysis [8–11], biomedical [12, 13], and Li-ion batteries [14]. The morphology and growth rate of NTAs can be easily controlled through optimization of anodization parameters, such as anodization voltage, time, and electrolyte composition [15–17]. The formation of TiO_2_ NTAs is a competition consequence between oxidation of titanium and electric-field-induced etching of TiO_2_ as well as chemical etching of TiO_2_ by fluoride ion [16, 18]. In the electrolytes, fluoride ions are believed to play an important role in the formation of nanotubular array, and three different fluoride-containing electrolytes have been developed. Dilute hydrofluoride acid (HF) aqueous solution is the first one used in anodization of titanium. However, the maximum thickness of TiO_2_ NTAs was limited to only several hundred nanometers due to the fast chemical dissolution of TiO_2_ NTAs by fluoride ions in aqueous solution [19]. Later, TiO_2_ NTAs with a thickness of a few micrometers were obtained in fluoride-containing buffer solution, in which the dissolution rate of formed TiO_2_ NTAs was reduced [20, 21]. Recently, a nonaqueous electrolyte composed of ammonium fluoride (NH_4_F) and a viscous organic electrolyte, such as ethylene glycol (EG) or glycerol was used, and the length of TiO_2_ NTAs was increased to several hundred microns [22–24].

To our knowledge, most of the previous works are focused on the fabrication of TiO_2_ NTAs on titanium foils. TiO_2_ NTAs on titanium foils have poor mechanical properties, that is, they are easily cracked and detached when subjected to stress on titanium foils [25]. In addition, when TiO_2_ NTAs on titanium foils are assembled into functional devices, the metal electrodes deposited on the oxide layer may diffuse into the titanium foil at elevated temperatures, which may cause an electrical short circuit. Most importantly, titanium foil underneath the oxide layer has thickness of 0.1–1 mm, making it incompatible for use in micro-electronic device [26, 27]. Thus, it is of great significance to form TiO_2_ NTAs directly on functional substrates [28]. So far, there are many reports about fabrication of TiO_2_ NTAs films on both conductive (ITO glass and Si) [29–31] and nonconductive (SiO_2_ and glass) substrates [27, 32–34]. These nanotubular structures were fabricated by anodizing titanium film under low fields (10–60 V). For example, Samira et al. [34] have fabricated ordered TiO_2_ NTAs on Si substrate under low field (40 V), where a long anodization time was required. Nevertheless, it is still a challenge to obtain high-quality TiO_2_ NTAs efficiently on Si substrate under high fields. Kim et al. [27] prepared highly ordered TiO_2_ NTAs by anodization of as-prepared titanium films which were deposited on patterned Si substrate. They also investigated the influence of the quality of titanium film and high anodization voltages (up to 200 V) on the morphology of TiO_2_ NTAs. However, the pre-patterning technique is limited because of its expense and the need for external treatments to achieve highly ordered TiO_2_ NTAs films. According to Ono et al. [35], the condition of high current density, i.e., a high electric field, is the key factor that determines the self-ordering of the pore arrangement.

Here, based on a high-field anodization technique we previously developed [36, 37], a 938-nm-thick TiO_2_ NTAs with high quality was achieved via anodizing titanium film on Si substrate at 120 V for 20 s, which is a significant improvement in growth rate. The effect of anodization time, applied voltage, and NH_4_F contents on the morphology and growth rate of TiO_2_ NTAs were systematically investigated. The current density measured over the entire anodization time was recorded, which can provide a way to understand the whole anodization process and estimate the termination time of anodization of titanium film. Finally, the effect of annealing temperatures on crystalline structure was examined.

## Experimental Details

### Ti Thin-Film Deposition on Si Substrate

The n-type Si (100) wafers were degreased by successively sonicating in acetone and ethanol for 10 min to remove contaminants followed by rinsing in deionized (DI) water, and then dried in nitrogen. Titanium film with a thickness of 360–530 nm was deposited on Si substrate by direct current (DC) magnetron sputtering in which titanium target with 99.999 % purity was used. The sputter chamber was pumped down to 4.0 × 10^−4^ Pa. Argon gas was purged into the chamber and maintained at 0.52 Pa during the deposition process. The Si substrate was kept at room temperature. The titanium film was deposited at 150 W for 10 min.

### Synthesis of TiO_2_ NTAs on Si Substrate

TiO_2_ NTAs were synthesized via an electrochemical anodization process, where a two-electrode configuration with graphite plate was used as cathode and titanium film as anode. The electrolyte was stirred at 800 rpm for heat dissipation during the reaction. A uniform local voltage was produced by a regulated DC power supply (Agilent Technologies N5752A). For the first set of experiment, the anodization time was varied from 2 to 120 s, whereas the anodization voltage was kept at 90 V and an organic electrolyte was kept invariant containing 0.3 wt% NH_4_F, 96 vol% EG, and 4 vol% DI water. For the second set of experiment, the anodization voltage was varied from 40 to 180 V, whereas the anodization time was fixed at 20 s and the electrolyte was kept the same. The above experiments were all performed at two temperatures of 20 and 0 °C. For the third set of experiment, NH_4_F content of the electrolyte was varied from 0.2 to 0.4 wt%, and the anodization was performed at 120 V for 15 s. The as-grown TiO_2_ NTAs on Si substrate were annealed at different temperatures (350, 550, and 750 °C). The details of the anodization parameters for three different sets of TiO_2_ NTAs are summarized in Table 1.

**Table 1:** Details of anodization parameters for three different sets of TiO_2_ NTAs

	Set (1) variation of anodization time	Set (2) variation of applied voltage	Set (3) variation of NH_4_F contents
Anodization time (s)	2–120	20	15
Applied voltage (V)	120	40–180	120
The contents of NH_4_F (wt%)	0.3	0.3	0.2–0.4

### Characterization

The morphologies of TiO_2_ NTAs were examined using field-emission scanning electron microscope (SEM, Zeiss Ultra Plus FESEM), equipped with an energy dispersive X-ray (EDX) analyzer. EDX measurements were conducted at 10 keV to analyze the elemental composition of TiO_2_ NTAs on Si substrate. The crystallinity of TiO_2_ NTAs was investigated by X-ray diffraction analysis (XRD, D8 Discover with GADDS, Bruker Advanced X-ray Solutions, Inc.). Raman spectra were measured using the 514 nm of an Ar + laser as the exciting light source (Jovin Yvon Labram 800002).

## Results and Discussion

### Formation of TiO_2_ NTAs on Si Substrate

**Fig. 1:**
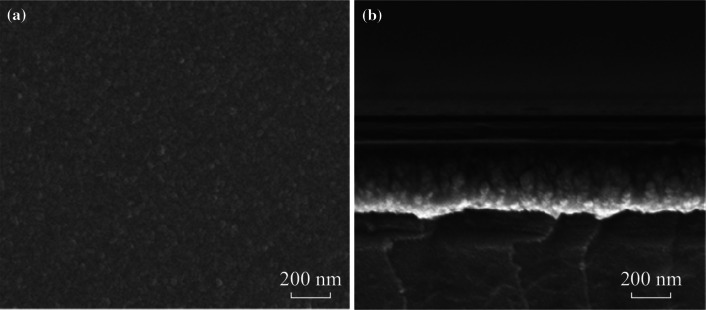
SEM images of *top* view (**a)** and cross section (**b)** of Ti film deposited by the direct current magnetron sputtering

**Fig. 2:**
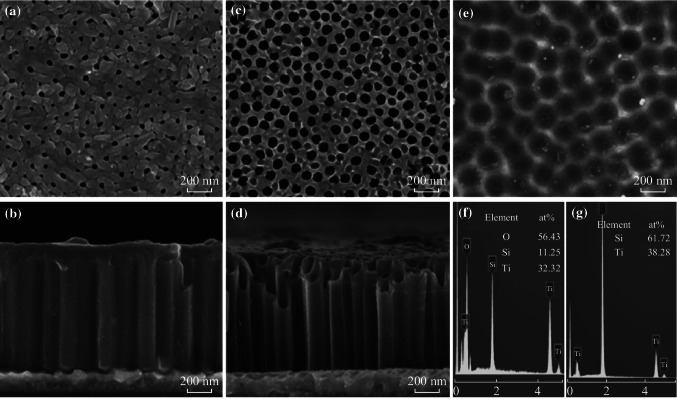
The *top* (**a)** and *side* view (**b)** of TiO_2_ NTAs on Si substrate formed at 150 V for 20 s. The *top* (**c)** and *side* view (**d)** of the sample immersed in a 0.05 wt% HF aqueous solution for 25 min. **e** The *top* view of the sample immersed in a 0.05 wt% HF aqueous solution for 40 min. **f**, **g** The EDX spectra of the sample shown in Fig. 2c, e, respectively

Figure 1 shows the top and side view of the titanium film. It can be seen from Fig. 1a that the top of the film is covered with good packing of particles, producing a dense and smooth film. A side view with high density is shown in Fig. 1b. Figure 2 displays the morphologies and corresponding EDX analysis of the as-anodized TiO_2_ NTAs fabricated at 150 V for 20 s. As shown in Fig. 2a, the surface is covered by pronounced grains, and the nontubular layers above the tubes can be observed from the cross-sectional image (Fig. 2b). In order to remove the grains on the surface, the prepared samples were immersed into a 0.05 wt% HF aqueous solution for 25 min, and the images of the immersed sample are presented in Fig. 2c, d. It can be seen, the grains disappear after the immersion process, whereas the tubular structure appears. The TiO_2_ NTAs have an average diameter of ~80 nm and a nanotube length of ~750 nm. When the immersion time was increased to 40 min, TiO_2_ NTAs peeled off from Si substrate completely, and only TiO_2_ NTAs remained (see Fig. 2e). This may be due to the chemical dissolution of the fluoride-rich TiO_2_ layer between the TiO_2_ NTAs and substrate when exposed to HF aqueous solution for a long time [38–40]. Therefore, all subsequent samples were immersed in a 0.05 wt% HF aqueous solution for 25 min to keep good adhesion of TiO_2_ NTAs on the substrate, even annealing at high temperature.

EDX measurements were conducted to analyze the elemental composition of TiO_2_ NTAs on Si substrate. Figure 2f shows the EDX spectra of the sample depicted in Fig. 2c, where some characteristic peaks of Ti, O, and Si appear. Figure 2g shows the EDX spectra of the sample displayed in Fig. 2e. Only Ti and Si peaks were observed, demonstrating that TiO_2_ NTAs were removed from the substrate completely after immersion for 40 min.

### Effect of Anodization Time on the Morphology of TiO_2_ NTAs

**Fig. 3:**
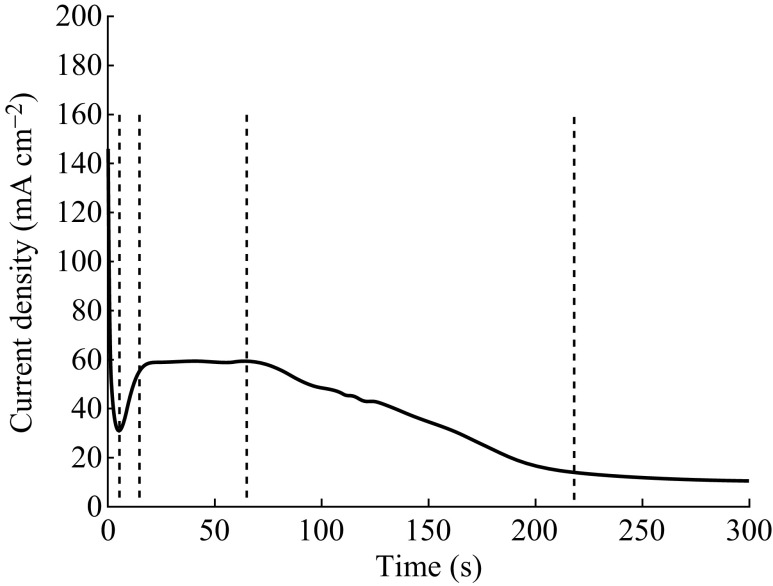
The *I*–*t* curve during the anodic growth of TiO_2_ NTAs at 90 V

**Fig. 4:**
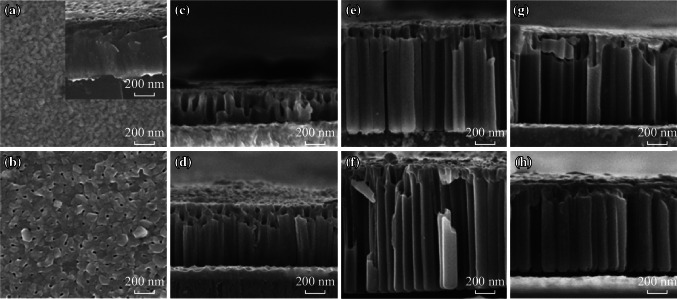
SEM images of TiO_2_ NTAs prepared at different anodization time **a** 2 s, **b** 7 s, **c** 10 s, **d** 20 s, **e** 40 s, **f** 60 s, **g** 90 s, and **h** 120 s

In order to better understand the anodization process, the current density–time (*I*–*t*) curve was recorded during anodization at 90 V (see Fig. 3). There are five stages in the anodization process. Firstly, the titanium film is oxidized under high field and a compact TiO_2_ barrier layer is formed (Fig. 4a). The high current density decays initially with increasing oxide thickness. Secondly, nanopores are formed as shown in Fig. 4b. These primary pores appear at the titanium grain boundaries due to the dissolution of the oxide layer, which causes a localized decrease in the oxide thickness and an increase in the current density. The third stage is a steady state established a few seconds later. This is due to the dynamic equilibrium between the titanium oxidation and electric-field-induced etching of TiO_2_ as well as chemical etching of TiO_2_ by fluoride ion. In this stage, the tubes grow longer with increasing time (Fig. 4c–f), whereas the thickness of the barrier layer is unchanged. As the anodization proceeds, the titanium film below the oxide layer is progressively consumed. Beyond a certain stage, the titanium film becomes discontinuous, creating highly resistant electric current pathways. Hence, the current density exhibits a decreasing trend in the fourth stage. Due to the vanishing of the titanium film, the corrosion of Si substrate and dissolution of TiO_2_ become dominant. The dissolution rate of TiO_2_ is faster than the oxidation rate of Ti, resulting in the decrease of TiO_2_ NTAs thickness (Fig. 4g, h). In the last stage, the current density reaches a steady state again. During this stage, there was no titanium film left for oxidation, and the current is attributed to the corrosion of silicon. Therefore, close monitoring of the current response during anodization can provide a simple method to determine the optimum anodization time.

**Fig. 5:**
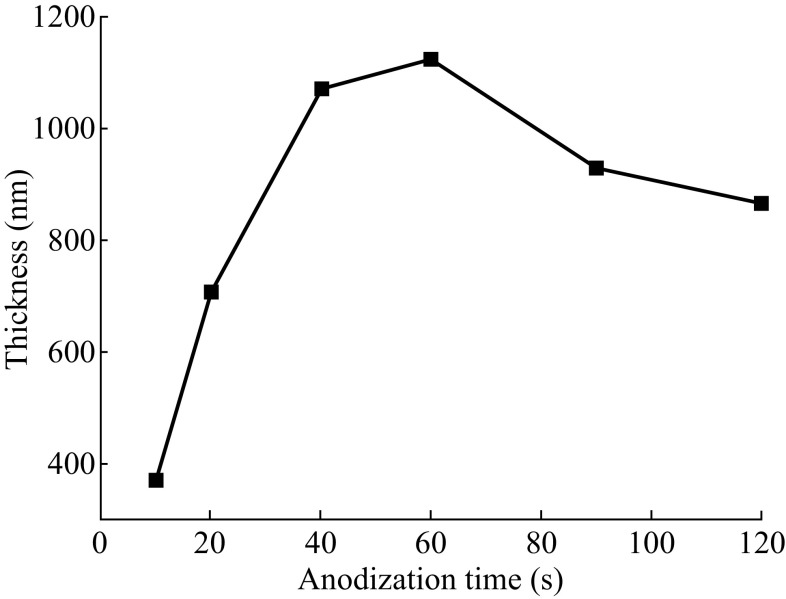
Dependence of the thickness of TiO_2_ NTAs formed at 90 V on the anodization time

Figure 5 illustrates how the length of TiO_2_ NTAs changes with the anodization time. The detailed SEM micrographs of TiO_2_ NTAs obtained at different anodization times are given in Fig. 4. After anodization for 2 s, no nanotubes are observed (Fig. 4a). After 10 s, the thickness of anodic film is about 370 nm, but the nanotubes are not well-defined (Fig. 4c). Nanotubes are clearly visible after 20 and 40 s (Fig. 4d, e), their length being about 700 and 1070 nm, respectively. It can be observed that the nanotube length is longer than the thickness of the deposited titanium film, which could be due to the volume expansion during anodization [32, 33]. When the anodization time was extended to 60 s, the anodic nanotubes become longer. But with anodization time increasing to 90 s, the length of TiO_2_ NTAs decreases to 930 nm (Fig. 4g). For longer anodization times, there was only little titanium film left. The oxidation of Si substrate and the dissolution of TiO_2_ NTAs become dominant, causing the decrease of TiO_2_ NTAs thickness. Despite the corrosion of Si, the mechanical adhesion of TiO_2_ NTAs was not affected. Even after sonicating for 10 min, the TiO_2_ NTAs exhibit good adhesion to the Si substrate (result not shown here).

### Effect of Applied Voltage on the Morphology of TiO_2_ NTAs

**Fig. 6:**
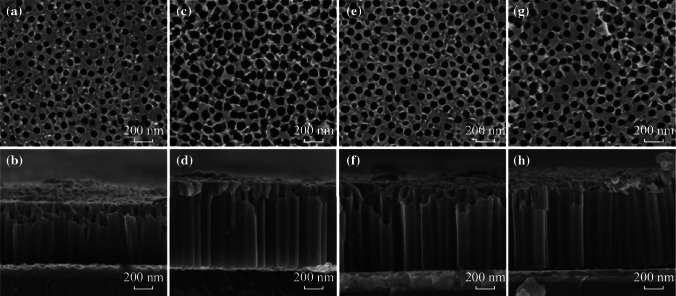
The *top* view of TiO_2_ NTAs grown at different anodization voltages for 20 s: **a** 90 V, **c** 120 V, **e** 150 V, and **g** 180 V. **b**, **d**, **f**, **h** The corresponding *side* view

**Fig. 7:**
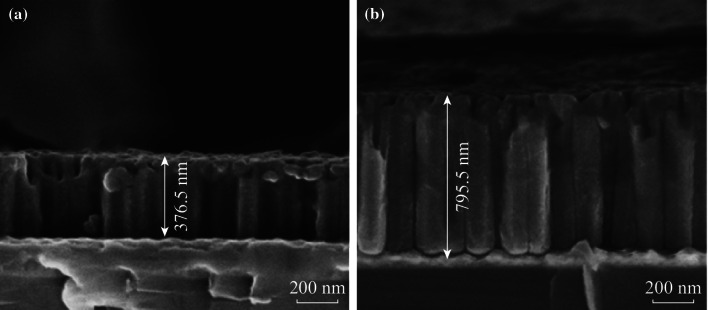
SEM micrographs of TiO_2_ NTAs fabricated at low fields for 150 s: **a** 40 V and **b** 60 V

Figure 6 shows the SEM images of TiO_2_ NTAs formed at high voltages (90–180 V), demonstrating the change in the average diameter and growth rate of nanotubes. As shown in top views (Fig. 6a, c, e, g), the pore diameter of TiO_2_ NTAs under different voltages ranges from 70 to 100 nm. TiO_2_ NTAs formed at 120 V have a larger pore diameter of ~100 nm, while TiO_2_ NTAs obtained at 90 V have a smaller diameter of ~70 nm. Figure 7 presents the side view of TiO_2_ NTAs under low fields (40 and 60 V). The growth rate (*R*) of TiO_2_ NTAs can be calculated using the formula *R* = h/t. Here, *h* means the thickness of the film, which can be measured from the SEM images (Fig. 6b, d, f, h, and 7), and *t* is the growth time. In our experiments, the growth rate of TiO_2_ NTAs under high fields is varied from 35 to 47 nm s^−1^, which is about 8 times faster than that under low fields (40 and 60 V). And TiO_2_ NTAs anodized at 120 V exhibit a maximum growth rate of ~47 nm s^−1^. The growth rate under different voltages is shown in Table 2.

**Table 2:** Growth rate of TiO_2_ NTAs grown via anodization under different voltages

	40 V	60 V	90 V	120 V	150 V	180 V
Anodization temperature (°C)	20	20	20	20	0	0
Anodization time (s)	150	150	20	20	20	20
Growth rate of TiO_2_ NTAs (nm s^−1^)	3	5	35	47	37	43

**Fig. 8:**
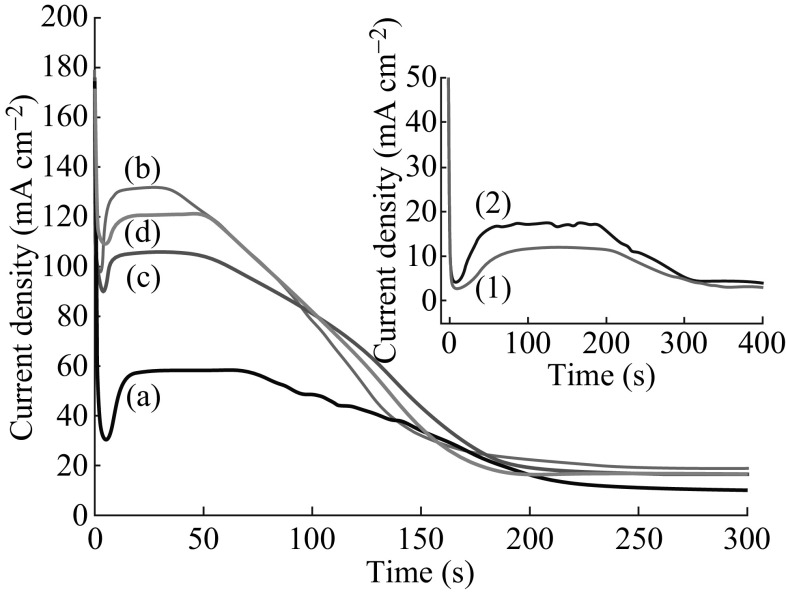
The *I*–*t* curves during the anodic growth of TiO_2_ NTAs under high voltages *a* 90 V, *b* 120 V, *c* 150 V, and *d* 180 V. The *inset* plots the *I*–*t* curves under low fields (*1*) 40 V and (*2*) 60 V. The experiments were carried out at two temperatures (20 °C for 40–120 V, and 0 °C for 150 and 180 V)

Figure 8 shows the *I*–*t* curves during the anodic fabrication of TiO_2_ NTAs under different voltages (90–180 V). The inset in Fig. 8 presents the *I*–*t* curves recorded under low fields (40 and 60 V). It can be found that the *I*–*t* curves reveal a similar changing trend. When anodization is performed at the same electrolyte temperature (20 °C), the steady-state current density increases with increasing applied voltages (40–120 V) (see Fig. 8 and its inset) [18, 41], and a higher current density leads to a higher growth rate, which is in line with the results shown in Table 2. However, there is a deviation at higher fields (150 and 180 V), which is attributed to the lower electrolyte temperatures (0 °C). During high-field anodization, it can produce much reaction heat, which is detrimental to the quality of TiO_2_ NTAs. Thus, at higher voltages (150 and 180 V), the electrolyte temperature was lowered to 0 °C. As mentioned above, TiO_2_ NTAs formation is a competition between the oxidation of Ti and field-enhanced dissolution of TiO_2_ as well as chemical dissolution of TiO_2_ by fluoride ions. Although the oxidation of Ti and electric-field-induced etching of TiO_2_ were enhanced at higher voltages, the mobility of fluoride ions in the viscous EG electrolyte was largely suppressed at lower temperatures (0 °C) [42], resulting in much lower chemical etching of TiO_2_ and a thicker barrier layer at the metal/oxide interface. During the anodization, the oxidation rate decreases as the barrier layer grows [43]. Consequently, TiO_2_ NTAs anodized at 150 and 180 V exhibit a lower current density than that anodized at 120 V. To check the reproducibility of the results, we have repeated the experiments conducted under different voltages (40–180 V) for three times. The average steady-state current density for each experiment has been listed in Table 3, which indicates that the result is repeatable.

**Table 3:** The average steady-state current density for each experiment conducted under different voltages

	Anodization temperature (°C)	Experiment times	Steady state current (mA cm^−2^)
40 V	20	1	11.6
		2	11.9
		3	11.7
60 V	20	1	16.7
		2	16.9
		3	17.0
90 V	20	1	58.2
		2	57.9
		3	58.3
120 V	20	1	131.4
		2	130.9
		3	131.0
150 V	0	1	104.9
		2	105.3
		3	104.9
180 V	0	1	119.9
		2	120.3
		3	120.1

### Effect of Fluoride Content on the Morphology of TiO_2_ NTAs

**Fig. 9:**
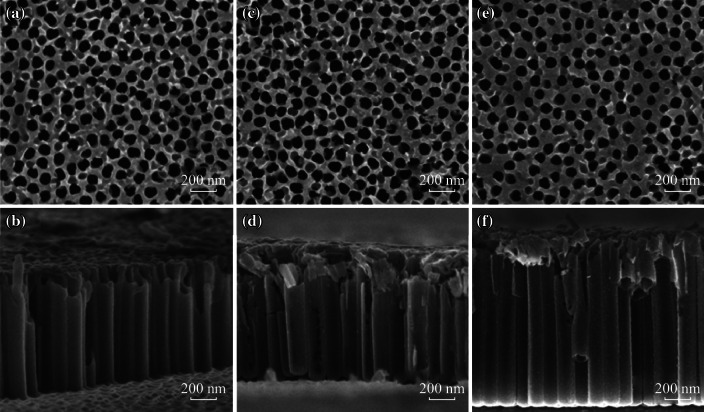
The *top* view of TiO_2_ NTAs obtained by anodizing in electrolyte containing different NH_4_F contents **a** 0.2 wt%, **c** 0.3 wt% and **e** 0.4 wt% NH_4_F. **b**, **d**, **f** The corresponding *side* view

In the electrolyte, fluoride ions play an important role in the nanotubular array formation. Figure 9 shows the SEM micrographs of TiO_2_ NTAs grown in electrolytes containing different fluoride contents (0.2–0.4 wt%). It can be seen that the pore diameters are varied from 85 to 95 nm, and no substantial change in pore diameter can be found with change in concentration (Fig. 9a, c, e). This result is in consistence with previous studies [18, 44]. Upon increasing the NH_4_F content, the length of TiO_2_ NTAs exhibits an increasing trend [18, 45, 46], ranging from 766 to 1285 nm (Fig. 9b, d, f).

**Fig. 10:**
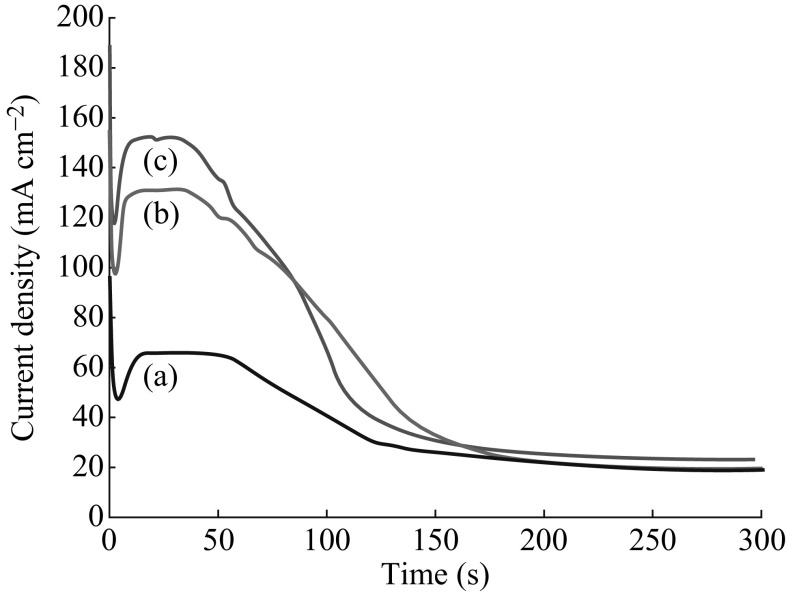
The *I*–*t* curves during the anodic growth of TiO_2_ NTAs at 120 V in electrolytes containing different NH_4_F contents **a** 0.2 wt%, **b** 0.3 wt%, and **c** 0.4 wt%

Figure 10 presents the *I*–*t* curves during the anodic growth of TiO_2_ NTAs in electrolytes containing different fluoride contents. It can be observed that the increase in fluoride concentration causes an increase in the recorded current density. Pena et al. have reported that the current density is a product of three different currents associated with the ion transport through the film (Ti^4+^, O^2−^, and F^−^) [24]. The increase in current density with higher fluoride content may be associated with an increase of the F^−^ transport. Since the current density is generally proportional to the growth rate, the fluoride contents have a significant impact on the growth rate, and a higher fluoride concentration leads to a higher growth rate, which is consistent with the above conclusions.

### Effect of Annealing Temperatures on Phase Structure

**Fig. 11:**
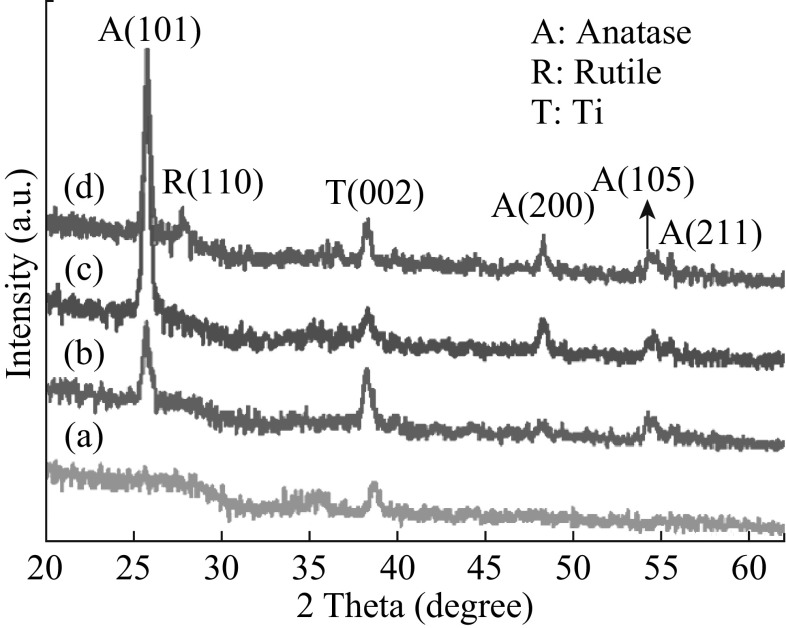
XRD patterns of TiO_2_ NTAs without heat treatment (**a**) or annealed at 350 °C (**b**), 550 °C (**c**), and 750 °C (**d**)

The change of crystalline structure of TiO_2_ NTAs annealed at different temperatures is shown in the XRD patterns (see Fig. 11). The XRD pattern of the pre-treatment sample (Fig. 11a) reveals that the nanotubular structure mostly consists of (002) Ti, indicating that the tubes are mostly amorphous prior to annealing. After annealing at 350 °C, typical peaks of anatase phase at 2*θ* near 25.3°, 48°, 54°, and 55° are observed in the XRD pattern (Fig. 11b), which correspond to the planes (101), (200), (105), and (211), respectively. For thermal annealing at 550 °C, the intensity of anatase peaks increases. It is evident that increasing the annealing temperature leads to an increase of crystallinity. For higher temperatures (750 °C), the rutile peak (110) at around 27.4° can be observed [47, 48]. With an increase of the annealing temperature, the Ti peak intensity decreases. Annealing in air supplements the oxidation of unanodized Ti and reduces the Ti peak intensity [33].

**Fig. 12:**
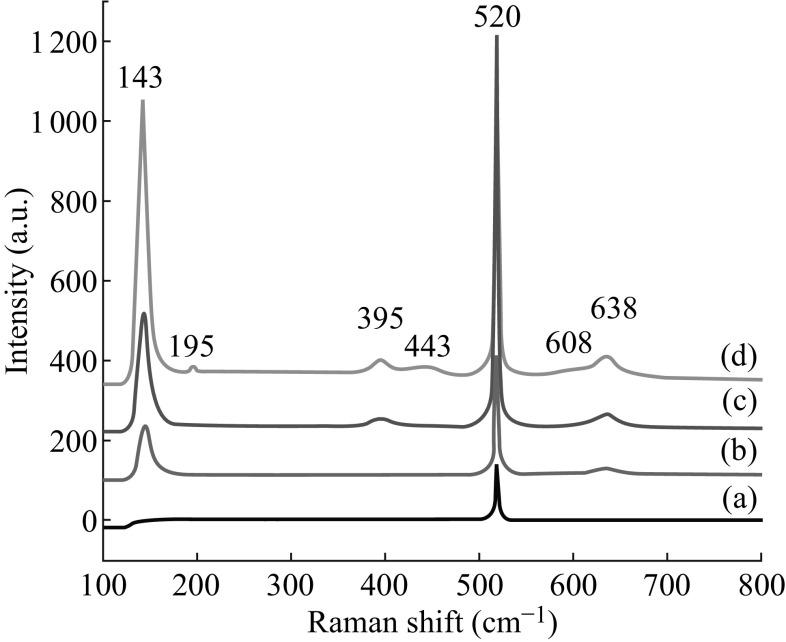
Room-temperature Raman spectra of TiO_2_ NTAs annealed at different temperatures **a** unannealed, **b** 350, **c** 550 °C, and **d** 750 °C

Raman spectra were employed to confirm the crystalline structure of TiO_2_ NTAs. The typical Raman spectra of TiO_2_ NTAs annealed at different temperatures are shown in Fig. 12. The Raman spectroscopy of unannealed samples (Fig. 12a) shows a broad spectrum, demonstrating that the unannealed sample is amorphous. When the sample was annealed at 350 °C, TiO_2_ NTAs exhibit specific peaks at 143, 395, and 638 cm^−1^, which are signatures of the anatase TiO_2_ [49]. With increasing the annealing temperature from 350 to 550 °C, the anatase TiO_2_ NTAs shows higher crystallinity. After annealing at 750 °C, Raman peaks observed at 443 and 608 cm^−1^ suggest that rutile is also present in the sample [50, 51]. The Si substrate signal can be identified by the Raman peak at 520 cm^−1^. This conclusion is in agreement with that observed from XRD patterns.

## Conclusion

In conclusion, highly ordered TiO_2_ NTAs have been obtained by high-field anodization of the Ti films deposited on Si substrate. It is found that the anodization voltage has a significant impact on the growth rate. A high voltage (90–180 V) leads to a high growth rate of TiO_2_ NTAs (35–47 nm s^−1^), which is nearly 8 times faster than the growth rate under low fields (40–60 V). When the applied voltage is 120 V, the anodization time is as short as 20 s for obtaining 938-nm-thick TiO_2_ NTA with high quality, which is a significant improvement in growth rate. Additionally, the fluoride content in the electrolyte will increase the length and growth rate, but has little influence on the pore diameter. Furthermore, the *I*–*t* curves measured over the entire anodization duration provide a facial method to monitor the anodization process and determine the optimum anodization time, leading to an easy obtaining of the desired nanotube arrays. Our work is anticipated to provide a way to fabricate highly ordered nanostructures on different functional substrates.
